# Formin homology domains of Daam1 bind to Fascin and collaboratively promote pseudopodia formation and cell migration in breast cancer

**DOI:** 10.1111/cpr.12994

**Published:** 2021-01-17

**Authors:** Leiyu Hao, Yan Liu, Xinqian Yu, Yuerong Zhu, Yichao Zhu

**Affiliations:** ^1^ Department of Physiology Nanjing Medical University Nanjing China; ^2^ Qinhuai District Nanjing Jinling Hospital Nanjing China; ^3^ State Key Laboratory of Reproductive Medicine Nanjing Medical University Nanjing China

## Abstract

**Objectives:**

Cancer cell migration to secondary organs remains an essential cause of death among breast cancer (BrCa) patients. Cell motility mainly relies on actin dynamics. Our previous reports verified that dishevelled‐associated activator of morphogenesis 1 (Daam1) regulates invadopodia extension and BrCa cell motility. However, how Daam1 is involved in actin filament assembly and promotes pseudopodia formation in BrCa cells remains unclear.

**Materials and methods:**

One hundred human BrCa samples were collected at Women's Hospital of Nanjing Medical University. Immunohistochemistry (IHC) was used to examine Daam1 and Fascin expression. Wound healing and Boyden chamber assays were used to explore cell migration and pseudopodia extension of BrCa cells. Co‐IP/pull down and Western blotting were performed to study the physical interaction between Daam1 and Fascin. Immunofluorescence assays were performed to observe whether Daam1 and Fascin were colocalized and mediated actin filament assembly.

**Results:**

Fascin was upregulated in BrCa tissues compared with that in paracarcinoma tissues. The downregulation of Fascin caused a decline in pseudopodia formation and cell motility. Moreover, we found that Daam1 interacted with Fascin via formin homology (FH) domains, especially the FH2 domain. Immunofluorescence assays showed that Daam1 and Fascin partially colocalized to actin filaments, and the knockdown of Daam1 or Fascin failed to colocalize to short and curved actin filaments.

**Conclusions:**

Daam1 specifically binds to Fascin via FH domains and cooperatively facilitates pseudopodia formation and cell migration by promoting actin filament assembly in BrCa.

## Graphical abstract

Daam1 notably collaborates with Fascin to promote the assembly of actin filament, pseudopodia extension and cell migration.

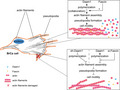

## 1 INTRODUCTION

Pseudopodia, including filopodia, lamellipodia and invadopodia, are temporary actin‐rich protrusions that drive the directed movement of living cells.^1^ Cell motility depends on the dynamics of actin filaments and pseudopodia, which are involved in adhesion to the extracellular matrix (ECM), guidance towards chemoattractants, degradation of ECM, transduction of extracellular signal, output of forces, etc.^2^, ^3^ We have demonstrated that dishevelled‐associated activator of morphogenesis 1 (Daam1), a member of formin family proteins, mediates ECM‐induced invadopodia extension and cell migration in breast cancer (BrCa).^4^ However, the precise role of Daam1 in the assembly of actin filaments and the formation of pseudopodia is still unclear.

Daam1 binds to the growing barbed ends and mediates filament actin (F‐actin) polymerization when actin filaments are elongating.^5^ Evolutionarily conserved formin protein Daam1 contains two highly homologous domains, formin homology domains 1 and 2 (FH1 and FH2).^6^, ^7^ FH1 is a rich profilin domain and mainly interacts with profilin actin molecules to supply FH2 with globular actin (G‐actin), while FH2 plays a key role in actin filament nucleation and elongation.^8^, ^9^, ^10^ It has been reported that active Daam1 enhances cancer cell motility, including BrCa, lung cancer, ovarian cancer, glioblastoma and osteosarcoma.^11^, ^12^, ^13^, ^14^, ^15^, ^16^, ^17^ Cell motility is an important foundation for tumour metastasis and invasion. Abnormal molecular biological activity of the actin cytoskeleton may strengthen or impair tumour cell motility.^18^

A crucial actin filament bundling protein Fascin is involved in tumour cell migration and invasion via modification of the actin cytoskeleton.^19^, ^20^, ^21^ High expression of Fascin is shown in ovarian tumours, BrCa, non‐small cell lung cancer, colon cancer, prostate cancer, etc, suggesting its oncogenic role in certain cancers mentioned above.^22^, ^23^, ^24^, ^25^, ^26^ In mammalian cells, Fascin is localized in filopodia and interacts with Daam1 to promote filopodia formation.^27^ Moreover, Daam1 accelerates actin assembly during mouse oocyte meiotic division through the alteration of Fascin expression.^28^ However, the underlying mechanism by which Daam1 associates with Fascin to regulate actin filament assembly, pseudopodia information and cell motility, especially in BrCa, is uncertain.

Here, we performed biochemical, immunofluorescent and immunohistochemical assays to reveal the interaction between Daam1 and Fascin and their roles in BrCa cell motility. Our results suggest that the binding of the FH domains of Daam1 to Fascin promotes the polymerization and bundling of actin filaments, which are required for pseudopodia formation and cell migration in BrCa.

## 2 MATERIALS AND EXPERIMENTAL METHODS

### 2.1 Clinical samples

A total of 100 BrCa samples were collected at Women's Hospital of Nanjing Medical University from 2019 to 2020. All patients had been diagnosed with BrCa by pathologists according to haematoxylin and eosin (H&E) staining. Ethical approval for the research was obtained from the Clinical Research Ethics Committee, Nanjing Medical University. Written informed consents were signed by all participants.

### 2.2 Cell culture

MCF‐7 and MDA‐MD‐231 cell lines were purchased from the Cell Bank of the Chinese Academy of Sciences (Shanghai, China). MCF‐7 and MDA‐MD‐231 cells were cultivated in Dulbecco's modified Eagle's medium (high glucose; REF 12800‐017, Gibco, USA) supplemented with 10% (V/V) foetal bovine serum (FBS) (catalog no. SH30396.03, HyClone, USA) and 1% penicillin/streptomycin (REF 15070‐063, Gibco) in a humidified incubator at 37°C with 5% CO_2_. Cell lines were confirmed monthly to be mycoplasma negative.

### 2.3 Plasmid constructions and transfections

Human Daam1 cDNA was obtained from our laboratory, and the full‐length fragment of Daam1 was amplified by PCR and inserted into the pcDNA 3.1‐3FLAG vector. Then, pcDNA 3.1‐3FLAG‐GBD‐FH1 (22‐600 bp), pcDNA 3.1‐3FLAG‐FH2‐DAD (601‐1068 bp) and pcDNA 3.1‐3FLAG‐FH2 (601‐984 bp) were generated by subcloning human Daam1 into pcDNA 3.1‐3FLAG. The cells were seeded in 24‐well or 60 mm plates (catalog no. 142475 or catalog no. 150462, Thermo) and cultured to 80%‐90% confluence. Then, the cells were transfected with plasmids (0.5 μg or 4 μg/well) using Lipofectamine 2000 reagent (0.8 μL or 10 μL/well) (REF 11668‐019, Invitrogen) in serum‐free Opti‐MEM (REF 31985‐070, Gibco; 35 μL or 200 μL/well). The cells were switched to fresh medium with 10% foetal bovine serum without penicillin/streptomycin for 6 hours after transfection and cultured for 24‐48 hours.

For gene knockdown, small interfering RNA (siRNA) duplexes specific for Fascin (On‐Target Plus: 5’‐GCUGUAUAAAGGCGUUAAUTT‐3’ and 5’‐GAGCUCAGAAUUGCAACAUTT‐3’; GenePharma, Shanghai, China) were transfected into MCF‐7 and MDA‐MD‐231 cells using Lipofectamine 2000 reagent as described above. Knockdown efficiency was assessed after transfection for 24 hours by measuring mRNA and protein levels in cell lysates using qRT‐PCR and Western blotting. Daam1‐GFP‐shRNA (kindly gifted from Dr Jie Mei, Wuxi People's Hospital affiliated with Nanjing Medical University) was transfected into MCF‐7 cells using Lipofectamine 2000 reagent and selected against puromycin. Clones stably expressing Daam1‐GFP‐shRNA were isolated. The cells were grown in the presence of puromycin (0.05 μg/mL; catalog no. 60210ES25, YESEN) and were maintained in a 37°C incubator with 5% CO_2_ as the parental cells.

### 2.4 Western blotting analysis

Cells cultured in 60 mm dishes were washed with PBS and then lysed with 2 × SDS sample buffer. The lysates were harvested, and abundant protein extracts were separated by 10% SDS‐PAGE. The following antibodies were used: anti‐Daam1 (1:1000 dilution; catalog no. 14876‐1‐AP, Proteintech), anti‐Fascin (1:1000 dilution; catalog no. 66321‐1‐lg, Proteintech), anti‐GAPDH (1:2000 dilution; catalog no. AC033, ABclonal), anti‐β‐actin (catalog no. AB21181, Bioworld), anti‐FLAG (catalog no. F1804, Sigma), polyclonal/monoclonal‐anti‐HA (catalog no. 923501/901501, Biolegend [Covance]) and anti‐His (1:1000, catalog no. 901501, Biolegend [Covance]) antibodies. Protein bands were tested after incubation with horseradish peroxidase‐conjugated antibodies and observed with High‐sig ECL Western Blotting Substrate (catalog no. 180‐5001, Tanon).

### 2.5 Coimmunoprecipitation

The cells were lysed with lysis buffer containing 50 mmol/L HEPES, pH 7.4, 100 mmol/L NaCl, 1 mmol/L EDTA, 1% Triton X‐100, 1 mmol/L phenylmethylsulfonyl fluoride and a 1% protease inhibitor mixture. Cell debris and unbroken cells were dislodged by centrifugation at 14 000 *g* and 4°C for 5 minutes. Clarified lysates were incubated with antibodies for 2 hours followed by partial protein A/G‐conjugated agarose beads (Pierce) (REF 20421, Thermo Scientific). After incubation for an extra 1.5 hours with agitation, beads were washed three times with lysis buffer and then lysed with 2 × SDS sample buffer. Samples were further subjected to Western blotting with anti‐Daam1, anti‐Fascin or anti‐FLAG antibodies.

### 2.6 In vitro pull‐down assay

His‐Fascin fusion protein was induced by IPTG for 2‐16 hours at 16°C and purified from BL21 bacteria. Next, the fusion protein was eluted three times from Ni‐NTA agarose beads by washing buffer (1 × PBS, 250 mmol/L imidazole, 1% Triton X‐100). For the binding assay, the cells transfected with FLAG‐tagged full‐length Daam1, FLAG‐tagged GBD‐FH1, FLAG‐tagged FH2‐DAD and FLAG‐tagged FH2 were lysed, and the supernatant lysates were extracted. Then, the lysate was incubated with His‐Fascin fusion protein and immobilized on Ni‐NTA beads (catalog no. 1018244, Hilden, Germany). After incubation, the beads washed three times on ice with ice‐cold lysis buffer were lysed with 2 × SDS sample buffer. Bead‐bound proteins were separated by SDS‐PAGE and analysed by Western blotting.

### 2.7 Immunohistochemistry (IHC)

One hundred BrCa pathological sections were deparaffinized at 55°C for 30 minutes. All sections were washed with xylene twice for 10 minutes each and then rehydrated by sufficient washes in 100%, 90%, 80% and 70% graded ethanol. Next, citrate buffer (pH 6.0; catalog no. P0083, Beyotime) was used to repair antigens at high temperature, and hydrogen peroxidase (0.3%; catalog no. P0100A) was applied to block endogenous peroxidase activity for 5 minutes. In addition, 2% BSA was used to block nonspecific antigens for 1 hour. The sections were incubated with primary anti‐Daam1 (1:50 dilution) antibody overnight. DAB and haematoxylin counterstaining were employed to visualize its expression. The expression of Daam1 and Fascin was determined by immunoreactivity score (IRS) equalling the percentages of positive cells multiplied by the staining intensity. The percentage of positive cells was scored as 0‐4:0 (<5%), 1 (6%‐25%), 2 (26%‐50%), 3 (51%‐75%) and 4 (>75%). The staining intensity was scored as 0‐3:0 (negative), 1 (weak), 2 (moderate) and 3 (strong). All immune‐stained sections were scanned by a microscope (Olympus Corporation, Tokyo, Japan).

### 2.8 Immunofluorescence (IF) and invadopodia staining

MCF‐7 cells seeded in 24‐well plates and invadopodia adhering to Boyden chamber membranes were fixed in 4% paraformaldehyde with PBS for 20 minutes. Then, cells and invadopodia were permeabilized and blocked by blocking buffer (saponin, BSA, fish skin gelatin, 10 × PBS, pH 7.4) for 30 minutes at room temperature. Next, to understand the extent of invadopodia, invadopodia on the lower sides of Boyden chamber membranes (3.0 μm pores) were stained with anti‐cortactin (1:1000 dilution, ab81208, Abcam) antibody for 1 hour at room temperature and then incubated with FITC‐labelled secondary antibodies (Sigma‐Aldrich) for 1 hour. To observe the colocalization state between the targeted protein and actin cytoskeleton, cells were seeded on glass slides and stained with TRITC‐labelled phalloidin (catalog no. 40734ES80, YESEN), anti‐Daam1 or anti‐Fascin for 1 hour and then treated with FITC‐labelled secondary antibodies (Sigma‐Aldrich) for 1 hour at room temperature. After washing with PBS, the cells were stained with DAPI and then fixed on glass slides with ProLong™ Gold antifade reagent (REF P36930, Invitrogen). The fluorescence images were captured through a fluorescence microscope (LSM710, Zeiss, Oberkochen, Germany) and analysed by ZEM software (Zeiss).

### 2.9 Wound healing assay

MDA‐MD‐231 cells were cultivated in 96‐well cell culture clusters. When grown to confluence, the cells were transfected with siRNA using Lipofectamine 2000 reagent for 6 hours, then switched to fresh DMEM with 10% FBS and further cultured for 24‐48 hours. Next, the cells were scratched artificially with a plastic pipette tip. After two rinses with PBS to remove cell residues, the wounded cellular monolayer was permitted to heal for 6 hour in DMEM containing 10% FBS.

### 2.10 Boyden chamber assay

MDA‐MD‐231 cells were plated into 24‐well cell culture clusters, and after growing to confluence, the cells transfected with siRNA using Lipofectamine 2000 reagent were switched into a Boyden chamber system (Coster, Corning, NY). The Boyden chamber included upper and lower chambers separated by a Matrigel‐coated polycarbonate membrane (8.0‐μm pore diameter; catalog no. 3422, Costar). The upper chamber membrane was covered with single‐cell suspensions (1 × 10^5^ cells) in serum‐free DMEM supplied with 5 μg/mL BSA, and the lower chamber was filled with DMEM with 10% FBS. The cells were allowed to migrate for 6 hours at 37°C. After that, the medium was discarded, the cells were washed with PBS and fixed with 4% paraformaldehyde in PBS. Then, the stationary upper cells were dislodged with a cotton‐tipped applicator, and the lower chamber membrane was stained with 0.5% crystal violet. The approximate number of cells crossed over the membrane was counted by a microscope (Olympus Corporation, Tokyo, Japan).

### 2.11 Statistical

All data were analysed using GraphPad Prism 8.0 software. Statistical analyses were primarily performed by one‐way ANOVA or Student's *t* test and are expressed as the means ± SD Differences were deemed significant if *P* values were <.05 (*), <.01 (**), <.001 (***) and <.0001 (****).

## 3 RESULTS

### 3.1 Knockdown of Fascin inhibits actin filament assembly and pseudopodia formation

**FIGURE 1:**
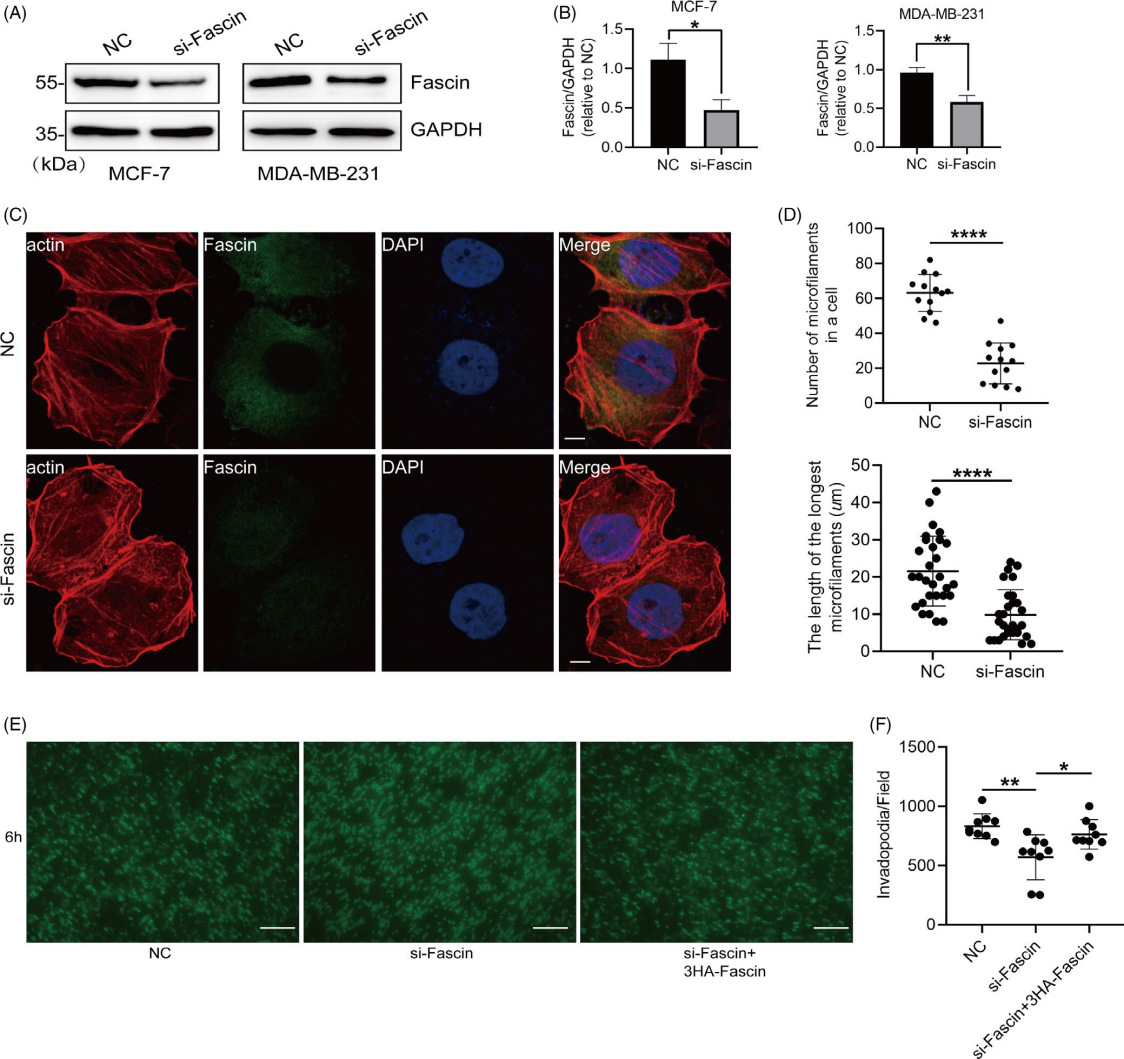
Knockdown of Fascin inhibits the assembly of actin filaments and the formation of pseudopodia. A and B, MCF‐7 and MDA‐MD‐231 cells were transfected with negative control (NC) and siRNA targeting Fascin (si‐Fascin). The expression of Fascin was determined by Western blotting. C and D, The morphology of actin filaments showed a short and curved state in Fascin‐knockdown MCF‐7 cells compared with NC cells. The number of actin filaments (length > 10 μm) and the longest actin filament in Fascin‐downregulated MCF‐7 cells were counted. Actin filaments were labelled with red fluorescently labelled phalloidin. Endogenous Fascin was incubated with anti‐Fascin antibody and green fluorescent protein secondary antibody. The nucleus was stained with DAPI (blue). Bar, 10 μm. E and F, MDA‐MD‐231 cells transfected with si‐Fascin and/or 3HA‐Fascin were detected for invadopodia formation in a 3.0 μm porous Boyden chamber. Invadopodia on the lower side of the membrane were treated with cortactin antibody and counted per field of microscope. Bar, 10 μm. Objective lens, magnification, ×40; numerical aperture, 0.95. *P < .05. **P < .01. ****P < .0001

To determine the effect of Fascin on the assembly of actin filaments in BrCa cells, we downregulated Fascin expression via siRNA interference and then observed the morphology of actin filaments. The expression of Fascin in MCF‐7 and MDA‐MD‐231 cells was reduced to ~50% by the overexpression of Fascin‐siRNA (Figure 1A,B). The number of actin filaments (length > 10 μm) and the longest actin filament in Fascin‐downregulated MCF‐7 cells were largely decreased compared with those in negative control (NC) cells (Figure 1C,D). To determine whether the low expression of Fascin inhibited pseudopodia (invadopodia) formation, MDA‐MD‐231 cells transfected with Fascin‐siRNA were plated onto the top of Boyden chambers with 3.0 μm pore membranes to induce pseudopodia formation. The downregulated Fascin significantly reduced the number of pseudopodia, which was rescued by the overexpression of exogenous Fascin (Figure 1E,F). Thus, the knockdown of Fascin inhibits the assembly of actin filaments and the formation of pseudopodia in BrCa cells.

### 3.2 Fascin is highly expressed in BrCa tissues, and downregulated Fascin suppresses the cell migration of BrCa

**FIGURE 2:**
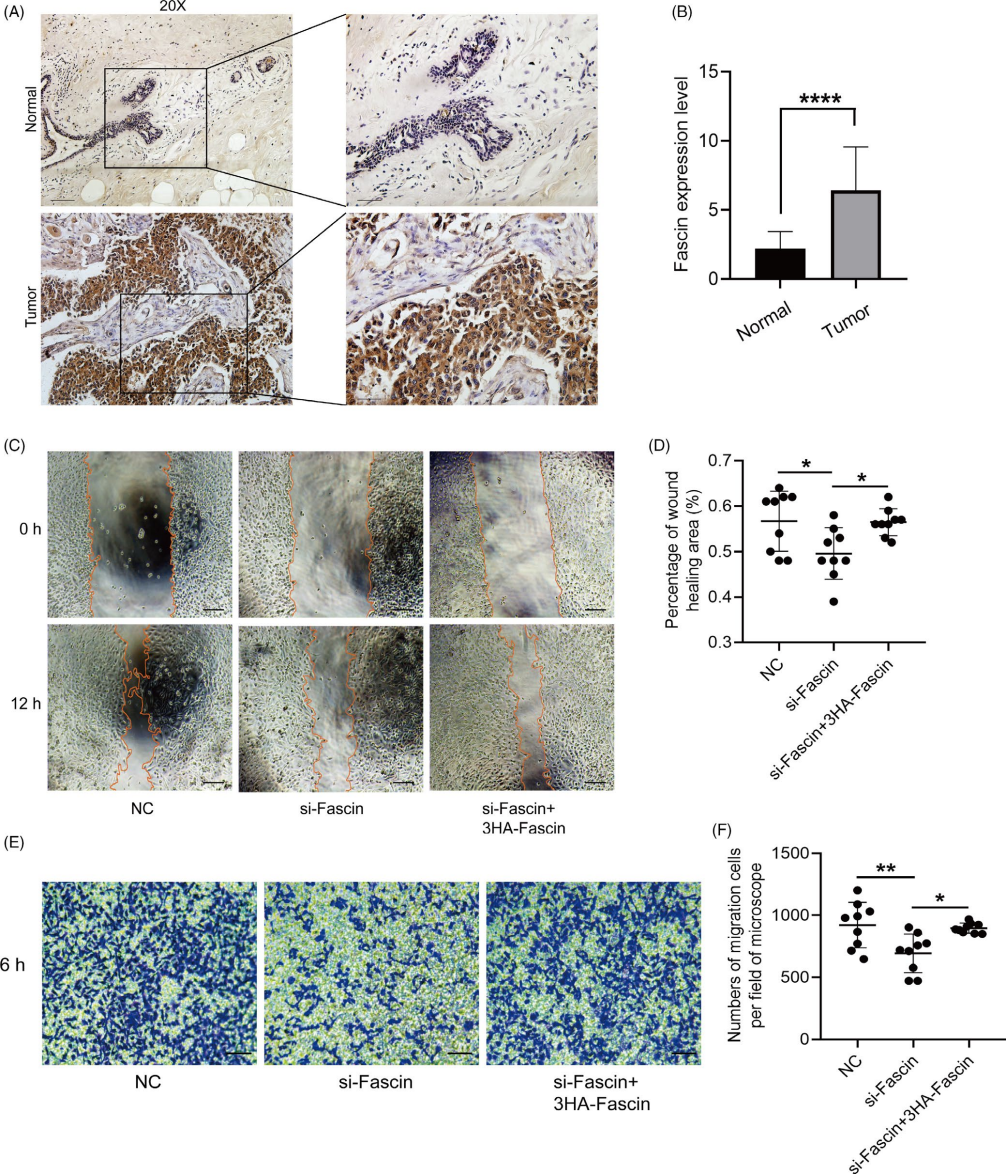
The expression of Fascin is upregulated in BrCa tissues, and downregulated Fascin restrains cell migration. A and B, In contrast to Fascin expression in paracarcinoma tissues, Fascin was highly expressed in BrCa tissues. Fascin was visualized with strongly positive staining in the cytoplasm. Bar, 500 pixel. Objective lens, magnification, ×20. The boxes were shown the local enlargement tissues. The immunoreactivity score (IRS) was calculated by multiplying the percentage of positive cells by the staining intensity. C and D, MDA‐MD‐231 cells transfected with siRNA targeting Fascin (si‐Fascin) and/or 3HA‐Fascin were allowed to migrate for 12 h. The wound healing area was measured and counted. Bar, 10 μm. Objective lens, magnification, ×20. E and F, MDA‐MD‐231 cells transfected with si‐Fascin and/or 3HA‐Fascin migrated for 6 h in Boyden chambers. The number of migratory cells per field was microscopically counted. Bar, 10 µm. Objective lens, magnification, ×20. *P < .05. **P < .01. ****P < .0001

After understanding the potential mechanism of the inhibition of actin filament assembly owing to the knockdown of Fascin, we further validated whether decreased Fascin mediated cell motility in BrCa. A total of 100 BrCa cases were enrolled, and Fascin expression was examined in clinical samples. These pathological sections were incubated with anti‐Fascin antibody via immunohistochemistry (IHC) assay. Fascin was highly expressed in BrCa compared with paracarcinoma tissues (Figure 2A,B). Also, we found that the expression level of Daam1 was associated with the status of lymph‐node metastasis of BrCa (*P* = .040, Table S1). For cell migration assays, we knocked down the expression of Fascin in MDA‐MD‐231 cells by siRNA interference and performed wound healing and Boyden chamber assays in vitro. We evaluated the areas of wound closure and found that Fascin‐decreased BrCa cells moved slower than control cells (Figure 2C,D). MDA‐MD‐231 cells transiently transfected with Fascin‐siRNA were seeded on Boyden chamber membranes (8.0 μm pores) and permitted to migrate for 6 hours. The number of migratory cells in the Fascin‐knockdown group was obviously diminished compared with that in the control cells (Figure 2E,F). Moreover, the overexpression of exogenous Fascin rescued the migration of Fascin‐silenced BrCa cells (Figure 2C‐F). Therefore, Fascin mediates cell migration in vitro and is highly expressed in BrCa tissues.

### 3.3 Daam1 interacts with Fascin and is highly coexpressed in the cytoplasm of BrCa cells

**FIGURE 3:**
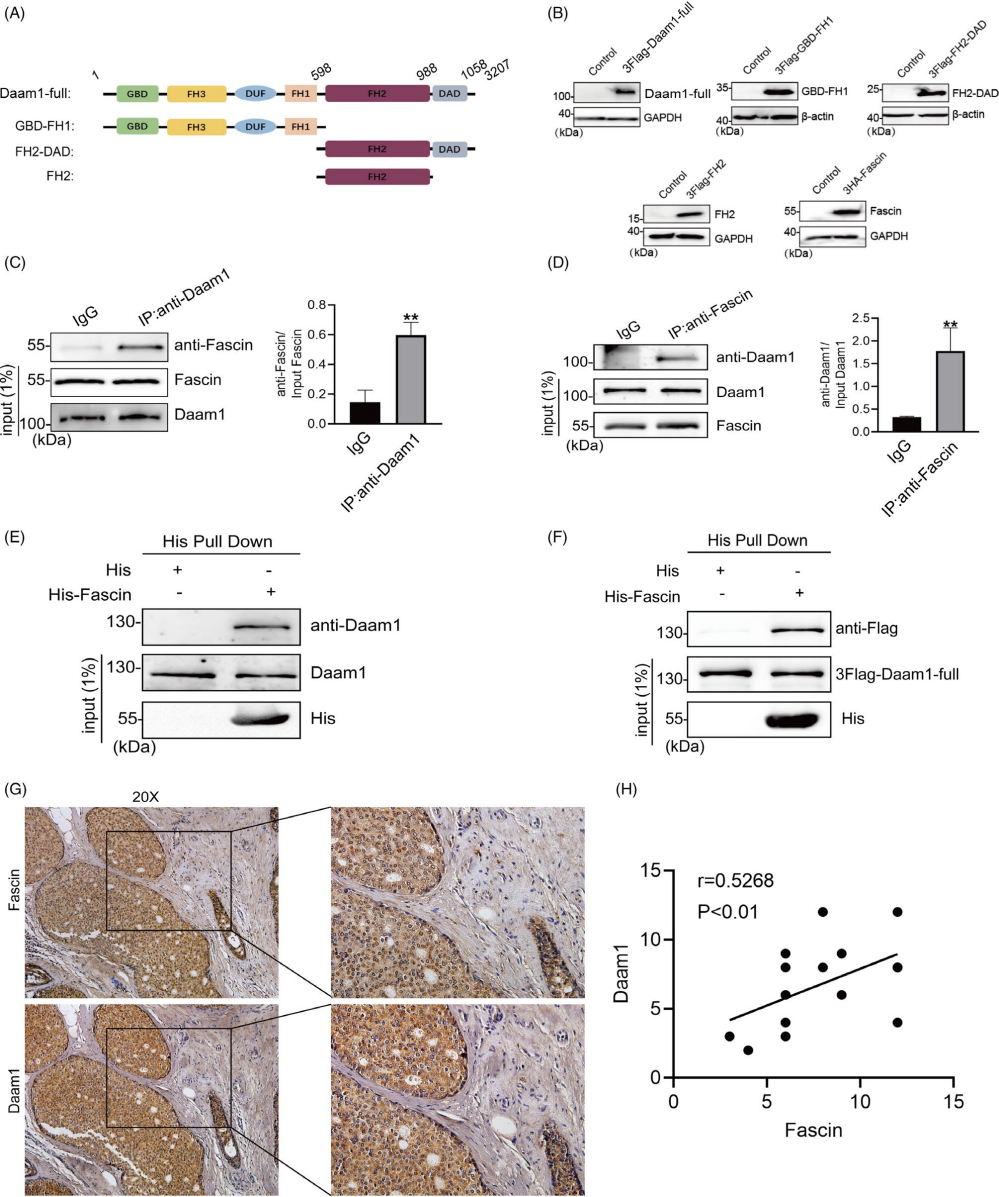
Daam1 interacts with Fascin and is significantly coexpressed in BrCa tissues. A, Schematic presentation of the domain structures of full‐length human Daam1 (Daam1‐full), GBD‐FH1, FH2 and FH2‐DAD. B, Daam1‐full, GBD‐FH1, FH2, FH2‐DAD and human Fascin were inserted into pCDNA 3.1‐3FLAG or pCDNA 3.1‐3HA vectors. The expression of exogenous proteins in BrCa cells transfected with the indicated constructs was examined by Western blotting. C and D, Endogenous Daam1 and Fascin coimmunoprecipitated with each other with the anti‐Daam1 or anti‐Fascin antibody. The whole‐cell lysates were analysed by immunoblotting with the indicated antibodies. **P < .01. E and F, For in vitro binding assay, MCF‐7 cells expressing endogenous Daam1 or transfected with 3FLAG‐Daam1‐full were lysed and then mixed with bacterially expressed His‐Fascin. The pull‐down products and all inputs were immunoblotted with the indicated antibodies. G and H, Daam1 and Fascin showed similarly strong positive staining in BrCa immunohistochemical sections. Bar, 10 μm. Objective lens, magnification, ×20. The coexpression relationship was analysed by correlation analysis. *r* = .5268, *P* < .01

Because of the role of Daam1 in pseudopodia dynamics and the effect of Fascin on the migration of BrCa cells, we investigated the physical interaction between Daam1 and Fascin. We constructed full‐length Fascin and a series of Daam1 truncated constructs and validated the expression efficiency of the indicated proteins by Western blotting (Figure 3A,B). MCF‐7 cells expressing endogenous Daam1 or Fascin were lysed, and cell lysates were immunoprecipitated with anti‐Daam1 or anti‐Fascin antibodies, followed by immunoblotting. Daam1 effectively interacted with Fascin in BrCa cells (Figure 3C,D). To test whether exogenous His‐tagged Fascin interacted with Daam1, an in vitro His pull‐down assay demonstrated that the bacterially expressed recombinant His‐Fascin bound to endogenous Daam1 (Figure 3E). Similarly, overexpressed exogenous 3FLAG‐tagged Daam1 notably interacted with purified His‐Fascin (Figure 3F). In addition, we studied whether Daam1 and Fascin were highly coexpressed in BrCa tissues. The immunohistochemical results showed that Daam1 and Fascin were significantly coexpressed in the cytoplasm of BrCa cells in pathological sections (Figure 3G,H). In summary, Daam1 remarkably interacts with Fascin.

### 3.4 Daam1 binds to Fascin via FH domains

**FIGURE 4:**
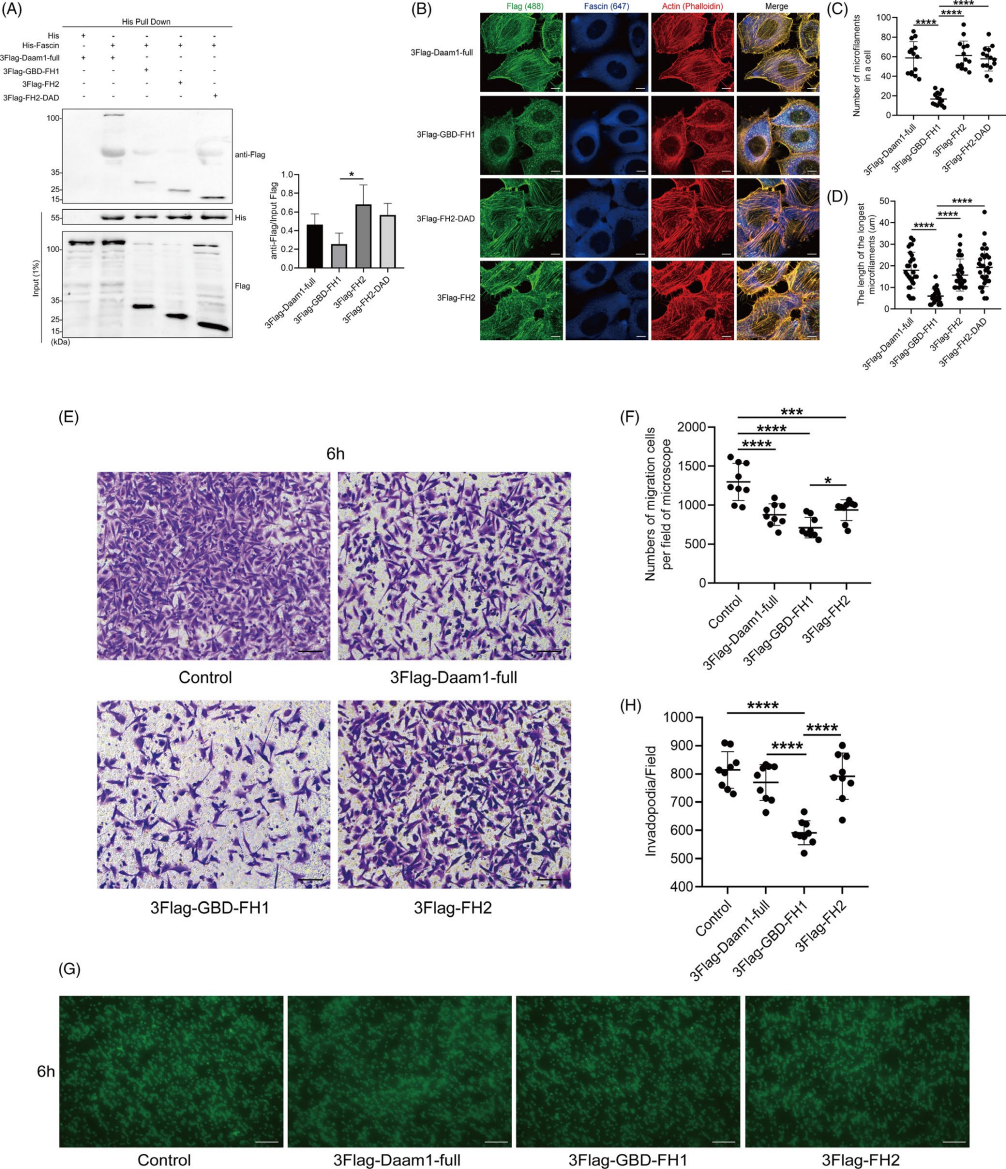
Daam1 binds to Fascin via FH domains. A, The lysates of MCF‐7 cells transiently expressing 3FLAG‐Daam1‐full, 3FLAG‐GBD‐FH1, 3FLAG‐FH2 and 3FLAG‐FH2‐DAD were mixed with bacterially expressed His‐Fascin and examined by immunoblotting with anti‐FLAG antibody. B‐D, The morphology of actin filaments in MCF‐7 cells expressed with the indicated constructs was visualized by immunofluorescence microscopy. The number of actin filaments (length > 10 μm) per cell and the length of the longest actin filaments were counted. Bar, 5 μm. E and F, MDA‐MD‐231 cells transfected with the indicated constructs migrated for 6 h in Boyden chambers. The number of migratory cells per field was counted. Bar, 10 μm. Objective lens, magnification, ×20. G and H, MDA‐MD‐231 cells were transiently transfected with the indicated constructs. Pseudopodia (invadopodia) were allowed to extend in 3.0 μm porous Boyden chambers. Invadopodia on the lower side of the membrane were incubated with cortactin antibody and counted per field of microscope. Bar, 10 μm. Objective lens, magnification, ×40; numerical aperture, 0.95. *P < .05. ***P < .001. ****P < .0001

To investigate which domain of Daam1 specifically interacts with Fascin, we transfected BrCa cells with certain Daam1 truncated domains, including GBD‐FH1, FH2‐DAD and FH2. His pull down with the bacterially expressed His‐Fascin fusion protein and 3FLAG‐Daam1‐full/GBD‐FH1/FH2‐DAD/FH2 expressed in cells was performed. Pull‐down assays and Western blotting showed that the FH domains (FH1 and FH2) remarkably interacted with Fascin, and the FH2 domain more strongly interacted with Fascin than the FH1 domain (Figure 4A). Immunofluorescence assays showed that the number and length of actin filaments in 3FLAG‐GBD‐FH1‐overexpressing cells were largely decreased compared with those in 3FLAG‐Daam1‐full, 3FLAG‐FH2‐DAD and 3FLAG‐FH2‐overexpressing cells (Figure 4B‐D). To further study the effect of the FH1 and FH2 domains on cell migration and pseudopodia formation, MDA‐MD‐231 cells transfected with truncated Daam1 constructs were seeded onto 8.0 and 3.0 μm porous Boyden chambers and allowed to migrate and extend pseudopodia, respectively. The overexpression of the FH2 domain significantly promoted cell migration and pseudopodia extension compared with the FH1 domain, suggesting a dominant‐negative role of the FH1 domain on pseudopodia extension and cell migration (Figure 4E‐H). In total, these results demonstrate that Daam1 binds to Fascin via the FH domain, especially the FH2 domain, to promote actin filament assembly, pseudopodia extension and cell migration.

### 3.5 Daam1 and Fascin recruit each other to promote the assembly of actin filaments

**FIGURE 5:**
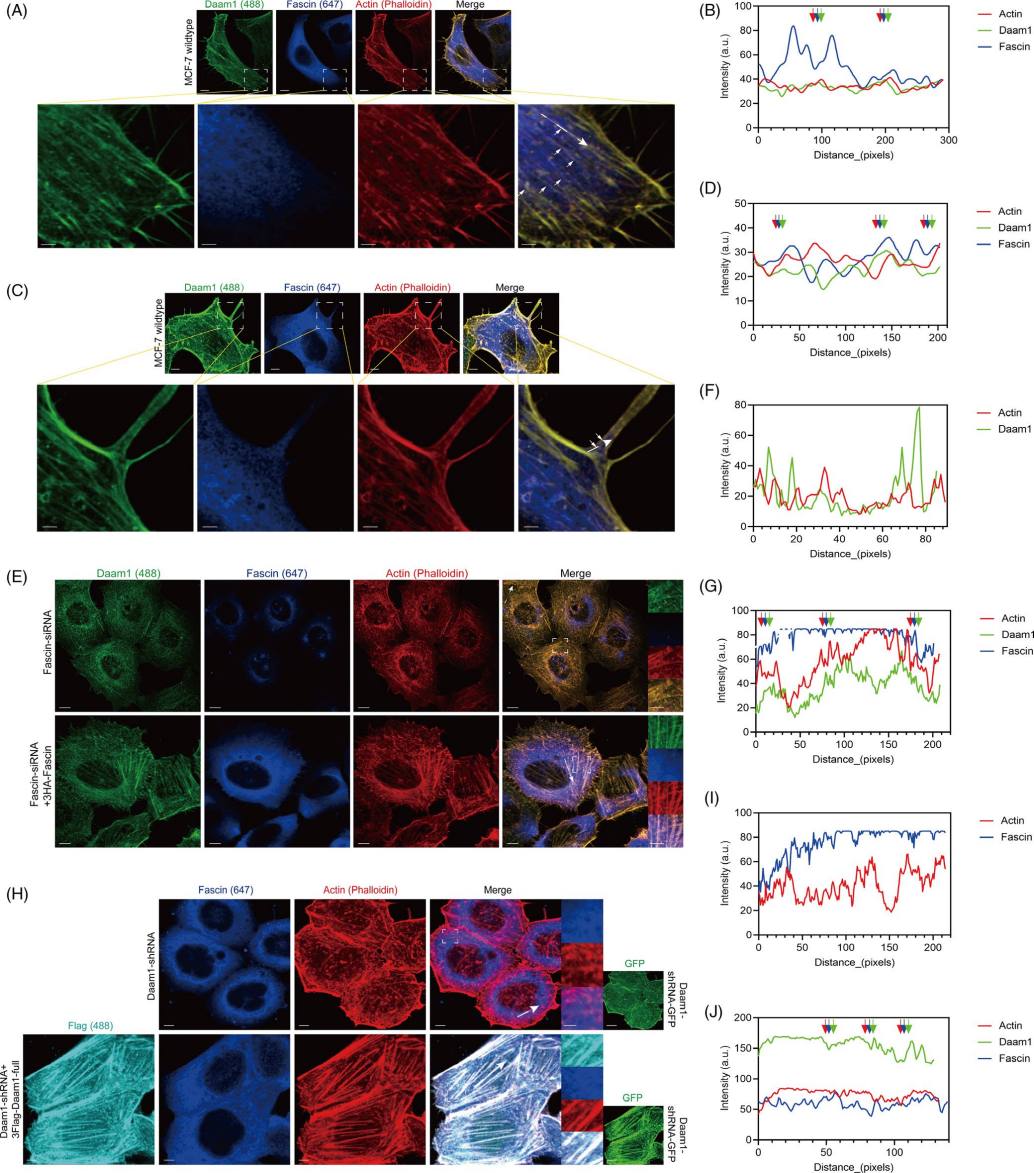
Daam1 and Fascin recruit each other, and they cooperatively mediate actin filament assembly and pseudopodia formation. A‐D, Endogenous Daam1 (green) and Fascin (blue) in MCF‐7 cells were colocalized with actin filaments and pseudopodia (filopodia, red). Bar, 5 μm. The boxed region shows the enlargement of local actin filaments, and white arrowheads indicate colocalization. Bar, 100 pixel. Fluorescence intensity profiles of Daam1, Fascin and actin were measured at the positions marked by arrows in merged insets. E‐G, Fascin‐silenced MCF‐7 cells (Fascin‐siRNA) were immunostained with antibodies against Daam1 (green) and Fascin (blue) and compared to the rescue group (Fascin‐siRNA + 3HA‐Fascin). The actin filaments were stained with phalloidin (red). Bar, 5 μm. The boxed region showed the local enlargement state. Bar, 5 pixel. H‐J, Daam1‐silenced MCF‐7 cells (Daam1‐shRNA) were immunostained with antibodies against Daam1 (cyan) and Fascin (blue) compared to the rescue group (Daam1‐shRNA + 3FLAG‐Daam1‐full). ShRNA targeting Daam1 was labelled with GFP (green), and actin filaments were labelled with phalloidin (red). Bar, 5 μm. The boxed region showed the local enlargement state. Bar, 5 pixel

**FIGURE 6:**
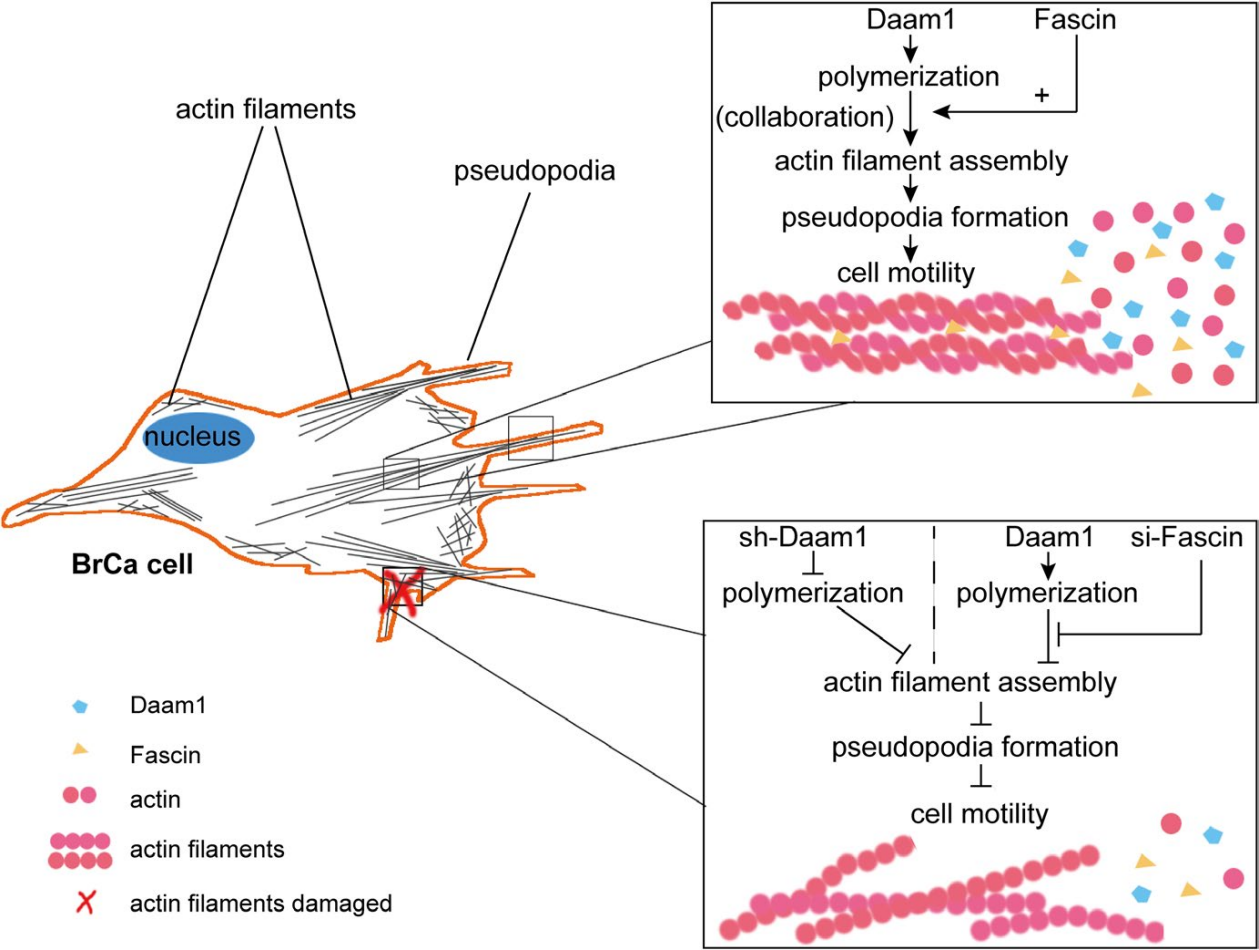
Schematic diagram of the mechanism by which Daam1 and Fascin collaborate to promote cell motility in BrCa cells

Having verified the physical interaction between Daam1 and Fascin, we studied whether Daam1 and Fascin were colocalized and mediated the assembly of actin filaments. Immunofluorescence assays showed that endogenous Daam1 and Fascin were colocalized with actin filaments (Figure 5A,B). Interestingly, endogenous Daam1 and Fascin were also colocalized in pseudopodia (filopodia; Figure 5C,D). Next, we investigated the role of Daam1 and Fascin in actin filament assembly. Knockdown of Fascin in MCF‐7 cells decreased the colocalization of endogenous Daam1 with actin filaments, and the puncta of Daam1 staining were diffuse (Figure 5E,F). Knockout of Daam1 in MCF‐7 cells disturbed actin filament assembly and weakened the colocalization of endogenous Fascin on actin filaments (Figure 5H,I). The overexpression of 3HA‐Fascin and 3FLAG‐Daam1 rescued actin filament assembly and the colocalization of Daam1, Fascin and actin filaments (Figure 5E,G,H,J). Therefore, these results indicate that Daam1 notably collaborates with Fascin to promote the assembly of actin filaments and cell motility (Figure 6).

## 4 DISCUSSION

Cancer cell motility is a central and key part of tumour metastasis. Although great effort and some research achievements have been made to explore the mechanism of BrCa metastasis, it remains a challenging research topic that needs continuous exploration.^29^ Our previous study revealed that active Daam1 acts as a downstream target of the Wnt5a/Dvl2 pathway, which is involved in Wnt5a‐induced BrCa cell migration,^11^ and integrin‐associated Daam1 stimulates pseudopodia (invadopodia) extension and cell haptotaxis in response to type IV collagen.^30^ Moreover, Fascin acts as an actin cross‐linker to mediate the elongation and bundling of actin filaments and invadopodia.^31^ Here, we identified that the FH domains of Daam1 are key sites that specifically bind to Fascin, especially the FH2 domain. Additionally, we uncovered that knockout of Daam1 or knockdown of Fascin failed to colocalize with actin filaments, resulting in damaged and shrunken cytoskeletons. These results demonstrate that Daam1 and Fascin notably interact with each other and collaborate to mediate actin filament assembly, which is required for pseudopodia formation and cell motility in BrCa.

In this study, we found that endogenous Daam1 and Fascin properly colocalized with actin filaments by immunofluorescence assay. After downregulation of the expression of Fascin, Daam1 did not colocalize with actin filaments and presented a cytoplasmic distribution. These results indicated that Fascin silencing caused the lessening of the assembled function of Daam1 for actin filaments and pseudopodia. Then, we examined the association of Daam1 and Fascin. The colocalization of Fascin on actin filaments was lacking in Daam1‐silenced BrCa cells. Similarly, Jaiswal *et al* also uncovered that Daam1 and Fascin collaborated to modulate perfect filopodial formation in mouse melanoma cells.^27^ Thus, we hypothesize that Daam1 and Fascin commonly mediate actin filament assembly and cell migration.

Accumulated evidence has confirmed that Fascin expression is notably increased in certain cancers, such as lung cancer, adrenocortical carcinoma and colorectal cancer.^32^, ^33^, ^34^ In this study, we also demonstrated that the expression of Fascin was significantly upregulated in BrCa compared with normal tissues. Downregulated Fascin expression dramatically retarded the assembly of actin filaments and pseudopodia formation, leading to a decrease in BrCa cell migration. In agreement with our viewpoints, live‐cell imaging techniques with the Fascin biosensor determined that Fascin dynamics dephosphorylated by FMNL2 governed rapid cytoskeletal adaptation and converted static filopodia to migrating tumour cells.^35^

Daam1 contains multiple domains that play various roles in cell biological functions.^36^, ^37^, ^38^ Among these domains, the FH2 domain plays an important role in actin filament nucleation and extension.^39^, ^40^, ^41^ Our study showed that Daam1 was associated with Fascin by FH domains (FH1 and FH2). The binding ability of the FH2 domain to Fascin was more powerful than that of the FH1 domain. Therefore, the physical interaction between Daam1 and Fascin was primarily determined by the FH2 domain. Some prior results have proven that the formin FH2 domain is able to interact with actin filaments. All‐atom molecular dynamics simulation analysis demonstrated that FH2 regulated this mechanism by interfering with subunit addition at the barbed end and supplying information about the equilibrium distribution of open and closed states.^42^ Next, we further investigated whether the FH2 domain has an impact on the migration of BrCa cells. FH2‐overexpressing BrCa cells migrated faster than cells transfected with other domains of Daam1. The number of pseudopodia in FH2‐overexpressing BrCa cells was greater than that in cells transfected with the GBD‐FH1 domain. These results indicate that the FH2 domain is the main site of Daam1 binding to Fascin.

In summary, this study offers a clear understanding of how Daam1 regulates the assembly of actin filaments to promote cell migration in BrCa. We show here that Daam1 and Fascin interact with each other and collaboratively govern cytoskeletal architecture and cell motility. Our data elucidate an underlying molecular mechanism that reveals an essential target for BrCa cell metastasis, which may be a promising target for the treatment of BrCa.

## CONFLICT OF INTEREST

The authors declare no conflict of interest.

## AUTHOR CONTRIBUTIONS

Yichao Z and LH designed this study. LH wrote the manuscript. LH, YL, XY and Yuerong Z prepared the figures. Yichao Z critically reviewed and edited the manuscript. All authors read and approved the final manuscript.

## Supporting information

Table S1

## Data Availability

The data that support the findings of this study are available from the corresponding author upon reasonable request.
