# Supplementary material for: Formin homology domains of Daam1 bind to Fascin and collaboratively promote pseudopodia formation and cell migration in breast cancer
Source: Cell Prolif. 2021 Jan 17;54(3):e12994. doi: 10.1111/cpr.12994 (PMC7941230; doi:10.1111/cpr.12994)
Supplement: Supplementary file 1 — Table S1 [file CPR-54-e12994-s001.docx]

**Supplement Table 1**

Relationships between Daam1 expression levels and clinicopathological traits of BrCa.

| Characteristics | Cases | Daam1 IRS^a^ | | χ^2^ | *P* value |
| --- | --- | --- | --- | --- | --- |
|  |  | Low | High |  |  |
| **Primary tumor** |  |  |  |  |  |
| T1 | 59 | 25 | 34 | 0.338 | 0.561 |
| T2-T4 | 41 | 15 | 26 |  |  |
| **Lymph-node metastasis** |  |  |  |  |  |
| N0 | 45 | 13 | 32 | 4.209 | **0.040** |
| N1-N3 | 55 | 27 | 28 |  |  |
| **ER status** |  |  |  |  |  |
| Negative | 36 | 16 | 20 | 0.463 | 0.496 |
| Positive | 64 | 24 | 40 |  |  |
| **PR status** |  |  |  |  |  |
| Negative | 45 | 17 | 28 | 0.168 | 0.682 |
| Positive | 55 | 23 | 32 |  |  |
| **HER2 status** |  |  |  |  |  |
| Negative | 79 | 33 | 46 | 0.492 | 0.483 |
| Positive | 21 | 7 | 14 |  |  |
| **Intrinsic subtypes** |  |  |  |  |  |
| TNBC | 20 | 8 | 12 | 0.000 | 1.000 |
| Others | 80 | 32 | 48 |  |  |

^a^ Low expression: IRS ≤ 4; High expression: IRS > 4.
